# Framing Helper Therapy to Support User Engagement: Causal Evidence from a Public Deployment of a Mental Health Support Text Messaging Program

**DOI:** 10.1145/3772318.3791591

**Published:** 2026-04-13

**Authors:** Tony Liu, Bhargavi Patil, Thu Ngo, Chris Karr, Theresa Nguyen, Rachel Kornfield, Jonah Meyerhoff

**Affiliations:** Computer Science, Mount Holyoke College, South Hadley, Massachusetts, USA; Mount Holyoke College, South Hadley, Massachusetts, USA; Mount Holyoke College, South Hadley, Massachusetts, USA; Behavioral Research Innovation Center, Chicago, Illinois, USA; Mental Health America, Alexandria, Virginia, USA; Preventive Medicine, Northwestern University, Chicago, Illinois, USA; Preventive Medicine, Northwestern University, Chicago, Illinois, USA

**Keywords:** Email/Texting/Communication, Health - Wellbeing, Digital Mental Health, Helper Therapy, Empirical Study, Quantitative Methods

## Abstract

Digital peer-to-peer mental health tools have shown promise in supporting the well-being of those receiving help and giving it (i.e. helper therapy), but promoting engagement remains a challenge. We examine whether the *framing* of helper therapy exercises motivates active user participation and how user characteristics shape differential effects of the framings in a publicly deployed interactive text messaging-based mental health program. Among 3,817 users randomized to different helper therapy framings, we find causal evidence that framings which emphasize helpng oneself increase written engagement rates as much as 4.6% over other framings, with even larger effects seen among minoritized identities. These self-focused framings also elicited messages with more positive, trust, and anticipation-related words and fewer fear, anger, disgust, and sadness words. Our findings highlight the importance of centering the user in the framing of digital intervention content, and personalizing digital mental health tools to align with a diversity of user identities.

## 1 Introduction

Over one in five adults in the United States experience mental illness, yet nearly half do not receive treatment [93]. While not a replacement for formal treatment, digital mental health (DMH) services can provide accessible, evidence-based resources and support programs to help address the treatment gap. The ubiquity of smartphones makes smartphone-based services a promising delivery modality for DMH interventions at scale, as they demonstrate efficacy in improving participant well-being within controlled research environments [27]. However, adapting smartphone DMH services from controlled settings to real-world use is challenging due to the difficulty in sustaining active engagement [8], especially when the interventions need to fit into the context of users’ daily lives [14].

Peer support represents a major pathway for DMH interventions to deliver help at scale, but also presents a substantial challenge in eliciting user participation. Peer support takes many forms in DMH interventions, including online support groups and forums [53], buddy systems or one-on-one chats [70], and systems that solicit and deliver crowdsourced suggestions, advice, and active listening [7, 22]. Such designs have potential to help not only the person receiving peer support but also the one delivering it. The “helper therapy principle” holds that acts of helping others can lead to personal growth, increased self-esteem, and articulating or endorsing self-relevant strategies for managing one’s own concern [76]. Yet, while potentially valuable, giving support is effortful and its benefits may not always be clear to the helper. Individuals with depression symptoms highlight that technology-delivered prompts are likely to be ignored, particularly when they are in states of low mood or energy [51]. As helper therapy exercises require substantial energy, they need to be appropriately motivated for the helper to actively engage with them.

Prior work suggests that brief rationales or descriptions of the potential benefits of using a DMH tool can help close motivational gaps and prompt action, allowing users to understand how an activity aligns with their values or goals [32, 49]. Yet, in the case of helper therapy, the best way to *frame* activities to improve motivation is unclear as there are two primary pathways that could be emphasized: that of helping others, and that of helping oneself. Framing refers to presenting the same information in different ways, including highlighting particular meanings, outcomes, or values [10, 25]. A large experimental literature shows that even small shifts in framing change how people interpret and act on information, from foundational gain versus loss studies in decision making [43, 94] to communication scholarship examining differential effects when messages emphasize causes versus solutions, individual responsibility versus systemic context, or emotional narratives versus factual information [10, 25, 36]. Similarly, helper therapy may be framed either as an exercise focused on personal benefits or aiding others, potentially affecting whether individuals engage and the linguistic properties of the messages they contribute, which may have implications both for message receivers and senders [75]. Furthermore, DMH services employing helper therapy framings need to consider user contexts and preferences as users may differ in which framings they are receptive to [12]. DMH interventions that respond to the specific needs of those with minoritized identities have shown some promise in reducing mental health disparities [14, 69, 80]. Thus, there is a need to understand which framings of helper therapy will more effectively engage users of DMH interventions, and how user characteristics differentially shape engagement.

To inform the design of messaging interventions that effectively motivate helper therapy DMH activities, we address the following research questions:

- **RQ1 - Describing User Participation.** How do users of a DMH tool engage with a helper therapy-motivated writing task?
- **RQ2 - Framing E**ff**ectiveness.** How does the framing of system messages around helping oneself or helping others motivate participation in a helper therapy exercise?
- **RQ3 - Heterogeneity of E**ff**ects.** How do user characteristics, including minoritized identities, shape the effect of message framings that emphasize helping oneself or others?
- **RQ 4 - Linguistic Properties.** What are linguistic characteristics of the responses to a helper-therapy motivated writing task?

To answer these questions, we analyze an interactive text messaging dialogue that explores different framings for a helper therapy writing task. The dialogue was deployed to 3,817 users as part of Small Steps, a free public text messaging service provided by Mental Health America [1]. While users were all afforded the same opportunities to engage in the helper therapy exercises, they were randomly assigned to different helper therapy framings. Specifically, one framing focuses on varying the *target peer* of the support message i.e., who the user is asked to have in mind while writing, while another framing varies the *rationale* motivating why participating in the writing exercise is beneficial. We analyze the effects of these variations on user engagement rates, and how different user characteristics may shape the effects of these framings on engagement. The randomization of helper therapy framings provides causal evidence of their effects on user engagement at scale in the context of a large, publicly deployed DMH service.

Our contributions in this work are as follows:

- We describe a digital mental health helper therapy exercise that asks users to write supportive messages to a peer as a form of self-support (Sections 3 and 5.1).
- We perform causal analysis (Section 4) of the effects of different helper therapy *framings* around the 1) target peer and 2) rationale for the exercise on user engagement in a real-world sample of 3,817 U.S. users (Section 5.2).
- We examine how these framings vary in their effectiveness on engaging users with particular demographic characteristics, providing evidence on how to potentially tailor future peer support DMH interventions (Section 5.3).
- We analyze the linguistic properties of the support message responses to see how the different helper therapy framings shift the language of responses (Section 5.4).

## 2 Related Work

Our work extends previous research in helper therapy peer support principles (Section 2.1) as well as efforts to design and tailor text message-based digital mental health (DMH) deployments to users’ contexts and identities (Section 2.2).

### 2.1 Helper Therapy and Peer Support Benefits for the Helper

As a field, HCI has increasingly focused on examining effects of providing peer support in addition to receiving it [71, 86]. While giving peer-to-peer support can be demanding and exhausting [40, 71, 86, 100], it can also be beneficial to helper, an idea that psychologist Riessman [76] proposed 60 years ago as the “helper therapy principle.” Considerable research has since supported this premise [48, 81], showing that assisting others in a range of contexts improves well-being and coping with personal challenges, particularly when help is well-received [60, 68] and when it satisfies the helper’s needs or expresses their values [20], with helpers showing improvement in their own substance use disorders [56, 78], post-transplant recovery [77], and psychological trauma [30]. Helping others may also allow individuals to motivate themselves. For instance, by providing suggestions and re-framings, helpers may shift their perspectives, reappraise their own problems, or develop and affirm a repertoire of relevant coping and self-management strategies [79, 85].

To leverage benefits of helping, many digital interventions connect peers for the exchange of social support, including through online support groups [74, 75], crowd-sourced cognitive reappraisal tools [65, 87], and active listening interventions [7]. Many such interventions invite participants to serve as both help-seeker and helper, sometimes alternating their role based on their needs [7, 35, 67]. In addition to potential benefits to both parties, digital approaches are highly scalable since they can draw on the contributions of many helpers.

While HCI research has increasingly engaged in design of digital peer support interventions [29, 70, 95], less is known about how to motivate engagement with helper therapy in digital settings, particularly when peer support tasks are effortful, such as helping peers challenge negative thinking [19]. A recent scoping review on digital tools for cognitive reappraisal [47] also highlights relevant gaps, including a lack of rigorous evaluation of how intervention designs help users and limited use of designs that prompt users to take on perspectives of others. We seek to address these gaps by examining how design choices can most effectively elicit engagement in digitally facilitated peer support interactions.

### 2.2 User Context in Digital Mental Health (DMH) Deployments

DMH interventions stand to increase access to mental health resources, particularly for those reluctant to seek formal treatment. This medium offers a flexible alternative that appeals to demographics traditionally marginalized in the mental health space such as young adults [49, 83]. Beyond the practical benefits of providing access to resources, the act of engaging in these systems itself can also be therapeutic and provide the foundation for users to develop self-management skills [13]. This has led to a substantial body of research that focuses on optimizing the design of such DMH systems. For example, O’Leary et al. [70] studied designs that incorporate peer-guided chats, Kornfield et al. [50] recommended that crowd-sourced chat topics be validated by experts, and Bhattacharjee et al. [14] advocated for systems that help users navigate situational disruptors that can affect long-term use of DMH tools. Personalizing intervention content to the targeted population’s linguistic patterns [84], and demographic and cultural diversity [9] can also be beneficial, as failure to account for users’ backgrounds can lead to negative misinterpretations of cognitive behavioral therapy (CBT)-based messages [12]. By aligning with user needs, DMH interventions have shown success in decreasing depressive symptoms [27, 61, 67], but more research is needed to understand their use in real-world settings.

Prior work examines the user context around supporting emotional regulation in online platforms. Users who helped others more as opposed to receiving more support had greater decreases in depressive symptoms [24]. Analyses of Koko, an online application that facilitates text-based emotional peer-support interactions, show that synchrony in the language between the sender and receiver predicted greater benefits of using the platform, demonstrating how shared peer characteristics can facilitate more effective support [23]. Here, we aim to understand how user characteristics and the *framing* of peer support impact engagement in digitally-mediated helper therapy.

## 3 Study Overview

### 3.1 Program Design: Small Steps SMS

We analyze a DMH helper therapy writing exercise deployed by Mental Health America, a national mental health advocacy organization, as part of the *Small Steps* program [1]. Co-designed with individuals with depression and anxiety symptoms who are seeking to understand mental health symptoms through self-screening [61], Small Steps is an eight-week, automated, rules-based text messaging program that delivers evidence-backed *dialogues* that help users practice a variety of strategies to self-manage mental health concerns through psychoeducation content and optional exercises [62]. As those engaged in self-screening are often early in their help-seeking process [52], the tool is designed to be relevant whether or not they have adopted or identified with particular clinical diagnoses [49]. The program combines push-based approaches with interactive messages that allow users to engage more deeply when they have sufficient motivation and ability. Users may opt out of the program at any time. We focus on the helper therapy dialogue delivered on day 4 of the program, which asks all users to write a supportive message for a peer. The support messages written are banked to be reviewed by clinicians for potential future sharing but are not sent to peer recipients in real time. The primary purpose of the exercise is to engage the user in the principles of helper therapy as a form of self-support.

### 3.2 Helper Therapy Writing Exercise

**Figure 1:**
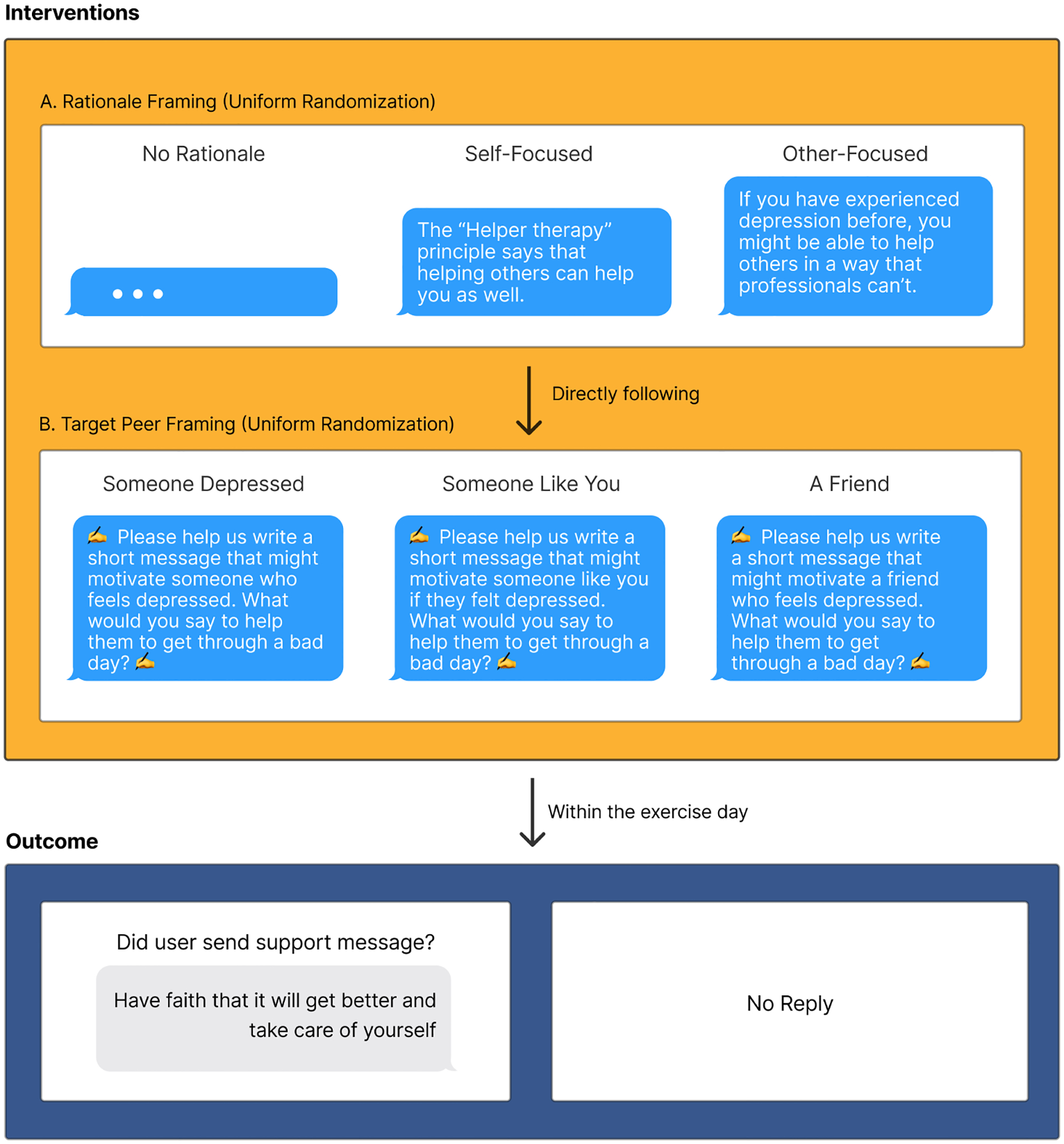
User flow for helper therapy dialogue interventions in the Small Steps program. Users receive two uniformly randomized framing interventions consisting of one of three rationales (A) followed by one of three peer support writing prompts (B). These messages occur on day four of the program. The primary outcome measurement is whether users write a support message responding to these prompts. Refer to Table A.1 for full text of the intervention messages.

In the helper therapy dialogue (Figure 1), there are two intervention points that frame the writing activity.

#### Rationale Framing.

The first intervention is a message that details the benefit of engaging in the writing exercise, which we call the “rationale framing” (Figure 1, A). The variations a user may see are as follows:

- No rationale is shown; the system continues to the prompt for writing a support message.
- The exercise is given a *self-focused* rationale: “helping others can help you.”
- The exercise is given an *other-focused* rationale: “you might be able to help others in a way professionals can’t.”

#### Target Peer Framing.

The second intervention is a message that asks the user to write a short support message, with variations on the “target peer” for whom the message is intended (Figure 1, B):

- The user is asked to support *someone who is depressed*.
- The user is asked to support *someone like them* who is depressed.
- The user is asked to support *a friend* who is depressed.

The program describes the peer as “someone who is depressed” to align with the most common mental health concern reported in Mental Health America’s screening data [2], which also frequently co-occurs with conditions such as anxiety, the symptoms of which are overlapping and often mutually reinforcing [44, 55]. Consistent with user preferences identified in related user-centered design work [49], the framing avoided clinical or diagnostic detail. Instead, it emphasizes a common transdiagnostic affective experience that is broadly relatable to users.

**Figure 2:**
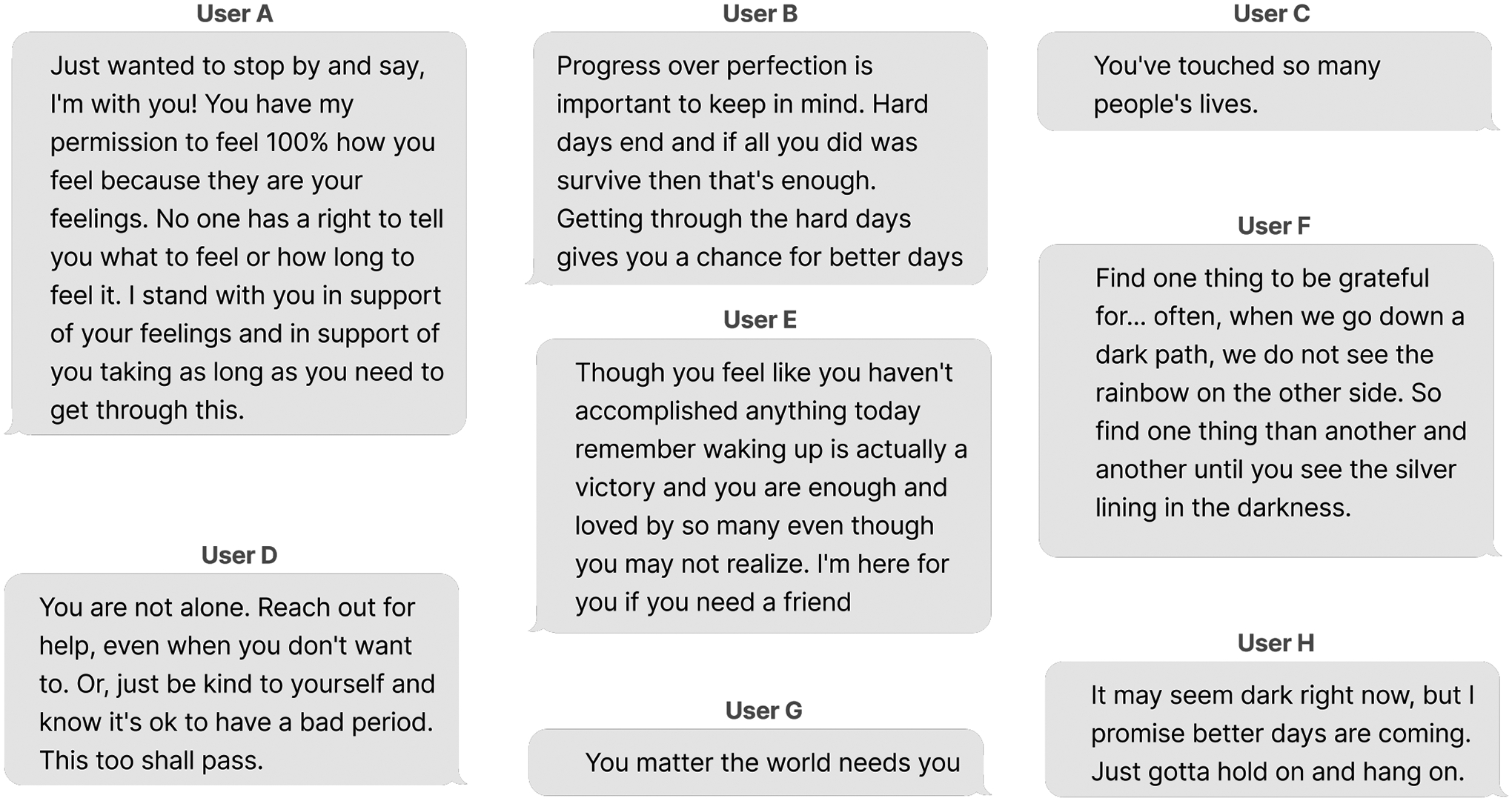
Example user support messages submitted for the helper therapy exercise across different individual conversations. Table A.2 contains the plain text of these examples.

Users experienced both the rationale and target-peer framings as part of the exercise. To quantify the impact of these framings on user engagement, we define our outcome of interest as whether or not the user responds to the writing prompt with a support message of their own (Figure 2).

### 3.3 Small Steps Users

Our study includes data from 3,817 Small Steps users who were sent the helper therapy exercise from April 2022 to December 2023. Users were invited to join the program upon completion of any of the available mental health screening tests provided through the Mental Health America website, such as those for depression or anxiety. Interested individuals completed an enrollment form, where they verified that they were a US resident, at least 18 years of age, and willing to share a mobile phone number. They were also asked to complete an optional survey that collected demographic information (Table 1) and included the Kessler Psychological Distress Scale (K10), a transdiagnostic measure of mental health distress [46]. K10 scores range from 10 to 50, with scores above 30 often considered likely to indicate severe psychological distress [3]. The sign-up process included informing users that their de-identified data would be used to understand system use and improve the program. The work presented here is a secondary analysis of this de-identified data and was conducted under a waiver of consent. The IRB at the authors’ institutions reviewed the protocol and determined that these analyses constituted quality improvement, not human subjects research.

## 4 Methodology

We describe how the helper therapy framing randomizations in the Small Steps program allow for valid causal inference (Section 4.1) and align with ethical considerations (Section 4.2), and describe subgroup analyses to explore how user identity may produce heterogeneity in these causal estimates (Section 4.4).

### 4.1 Randomization for Causal Effect Estimation

Randomization enables credible estimation of causal effects by removing the influence of *all* potential confounding factors from a study design [39]. Thus, our analysis of the helper therapy exercise leverages the existing uniformly randomized assignment of the *target peer* and *rationale* framings within the Small Steps deployment, ensuring that there are no significant variations in user characteristics between the target peer framing (Table A.3) and rationale framing (Table A.4) assignments.

### 4.2 Ethics of Randomization

While randomization can robustly establish causality, it must be implemented with ethical and equitable design to minimize potential harms. In particular, the Small Steps SMS deployment ensured *equipoise* of its helper therapy framings [31, 59] that is, whether there is genuine uncertainty about which framing is most beneficial. The deployment also considered the *fair distribution* [26] of any potential benefits of participating by providing all users equivalent opportunities to engage in the helper therapy writing exercise.

#### Equipoise.

The Small Steps SMS deployment approached the design of each variation of the helper therapy framing with the intent of evaluating which would be most beneficial for user participation. Thus, unlike typical randomization settings, we do not analyze any of the framings as a “control” intervention, as all of the intervention framings could have potential benefits. For the *rationale* randomization, users may either find the self-focused or other-focused motivations particularly compelling, or may prefer the absence of any additional rationale context from a notification overload perspective as it is one fewer system text message being sent. For the *target peer* intervention, users may resonate more with writing for “a friend” or for “someone like them,” or they may find it easier to write for a more generic “person who feels depressed” that provides some distance from their own personal context.

#### Fair distribution.

Because users of Small Steps voluntarily sign up for the program seeking mental health self-support, it is imperative that they all have access to the same program materials. Here, none of the randomization variations withhold any program services, and all users have equivalent opportunities to write support messages regardless of the framings they were presented.

### 4.3 Causal Interpretation of Statistical Analyses

Through randomization of the helper therapy framings, the mean difference in engagement rates between different framings can be interpreted as the average *causal contrast* between the two framings [37]. As a specific example, we can ask the causal question:

On average, how much does a target peer framing *T* of *someone like them* vs. *a friend* affect the likelihood a user writes a support message *Y*?

Under randomization, this causal quantity can be estimated as:

(1) P(Y=1|T=someonelikethem)−P(Y=1|T=friend)

Where *Y* = 1 indicates that a user wrote a support message. These causal questions can be formalized across all options within both the target peer and rationale framings. We use a two-proportion z-test for this difference in engagement rates to compute p-values and confidence intervals, which is an appropriate test due to randomization leading to independent samples for each framing.

We perform pairwise comparisons between all three options for both the *target peer* and *rationale* framings. This approach enables us to comprehensively evaluate the efficacy of the framings. Pairwise comparisons are recommended for multi-option randomization if the goal is focused evaluation of the intervention comparison (at the cost of multiple testing statistical corrections) [42]. We use Benjamini-Hochberg multiple testing correction applied within each set of interventions to control the false discovery rate [11].

### 4.4 Subgroup Analysis

In addition to estimating average causal effects, we analyze how user characteristics can shape the effects of the helper therapy framing interventions. Across scientific disciplines, there has been an increasing focus on heterogeneous effect estimation for subgroups, as many interventions (e.g., a public policy, a behavioral nudge, or a drug) have variable effects on different individuals, and studies that do not consider such variation run the risk of overlooking the needs of minority groups [17, 45]. In the context of digital mental health, there is considerable need for evidence-based design of services tailored to marginalized identities, as these groups can have higher barriers to accessing care and poorer mental health outcomes [9, 80]. To this end, we analyze the heterogeneous effect of helper therapy framings on engagement outcomes for Black, Indigenous, and People of Color (BIPOC) users as well as Lesbian, Gay, Bisexual, Transgender, and Queer or Questioning and More (LGBTQ+) users (full definitions in Appendix A.1), two groups in need of accessible and tailored resources [2, 73]. For example, we can modify our earlier causal question:

For LGBTQ+-identified individuals, how much does a target peer framing *T* of *someone like them* vs. *a friend* affect the likelihood of writing a support message *Y*?

Under randomization, this subgroup causal effect translates to [4]:

(2) P(Y=1|T=someonelikethem,X=1)−P(Y=1|T=friend,X=1)

Where *X* = 1 indicates that a user identifies as LGBTQ+. We note that this quantity describes effects within *X* = 1 and does not test for differences between subgroups. Instead, this formulation allows us to focus on effects within a particular user group to better understand which framings best encourage their engagement with the helper therapy writing exercise.

## 5 Results

Here we describe the quantitative results analyzing how different framings impact user engagement and language used within the helper therapy writing exercise.

### 5.1 RQ1: Describing User Interaction with the Helper Therapy Exercise

Overall, roughly one in five users (19.5%) send a support message (Table 2). Within the LGBTQ+ and BIPOC subgroups, rates are slightly higher but comparable to the full sample. Among all users who respond, their messages have a mean length of 170.8 characters and 34.2 tokens, with similar lengths and token counts in the LGBTQ+ and BIPOC subgroups. These message lengths are longer than what has been reported for typical everyday SMS [6, 57] and for open-ended psychological questionnaire responses via SMS [98]. The response engagement rates are in line with previous studies of more granular “per-exercise” engagement in DMH text messaging interventions deployed in real-world settings [5], particularly the 17% daily usage of peer-support apps [7], and motivate understanding whether different framings can encourage more user engagement with the helper therapy exercise.

### 5.2 RQ2: Causal Effects of Helper Therapy Framings on Engagement

We next measure the causal effects of the “target peer” and “rationale” framings on engagement in the helper therapy exercise. In the full study sample, the self-focused rationale increases support message engagement rates by 4.6% (95% CI [1.6, 7.7]) verses other-focused rationale, a statistically significant difference after multiple comparisons correction (Table 4). We also see that the self-focused rationale has directionally higher engagement rates versus no rationale, however this does not reach statistical significance.

### 5.3 RQ3: Subgroup Analysis

We examine how LGBTQ+ and BIPOC identities may shape differential causal effects of the helper therapy framings on engagement. Among LGBTQ+ users, the self-focused rationale framing increases engagement rates by 8.6% (95% CI [2.5, 14.5]) and 8.1% (95% CI [2.1, 14.1]) over the other-focused framing and no rationale being provided, respectively (Table 4). Among BIPOC users, we find that the “someone like you” target peer framing increases message writing rates by 9.5% (95% CI [3.4, 15.5]) over writing to support “someone depressed.” While not statistically significant, we also see directionally consistent increases in engagement rates of the “someone like you” framing over the “friend” target peer option. We observe substantial heterogeneity in the size of these effects within different demographic populations, underscoring the need for tailoring to support engagement of DMH interventions among traditionally underserved populations.

### 5.4 RQ4: Linguistic Properties of Peer Support Messages

We also sought to understand the language of users’ support messages. To do so, we computed the relative frequencies of words in the support messages from two lexica: the Linguistic Inquiry and Word Count (LIWC-22) lexicon widely used in psychological research [16] and the National Research Council Word-Emotion Association Lexicon (EmoLex), a commonly used English word dictionary mapping to basic emotions and sentiment [64]. For the LIWC lexicon, we focus on a subset of hierarchical categories that we apriori considered relevant to peer support writing (Appendix A.2 contains the full list of LIWC categories) and analyze total language used across support messages. Of note, we find that 7.35% of the total words are second-person pronouns compared to 3.71% of words being first person-pronouns, suggesting higher levels of peer-centered support language [18]. We also find that 9.31% of the total words have a present-focused time orientation compared to 2.06% of words with past-focused orientation and 2.9% of words with future-focused orientation, suggestive of users helping the peer they are writing for anchor to the present moment. Per-message aggregation of LIWC categories closely agrees with these findings (Table A.6).

To understand shifts in emotion language between the “target peer” and “motivation” peer support framings, we follow best practices in emotion lexicon inference [63] and focus on comparative analysis of EmoLex frequencies between the interventions. We aggregate support message texts across the intervention framings to maintain the causal interpretation of the results at a per-framing level and perform the same pairwise comparisons between intervention options as when we analyze engagement rates (see Appendix A.2 for additional details). While there are no statistically significant differences in emotion words among the “rationale” framings (Table A.8), within the “target peer” framing (Table 6) we find statistically significant increases in positive, anticipation, and trust words when comparing the “someone like you” target peer to the more general “someone depressed” target peer. Conversely, we see statistically significant decreases in fear, sadness, disgust, and anger words when comparing “someone like you” to “someone depressed.” As a robustness check, we also analyze these differences aggregating per-message among responders, which are directionally consistent with the per-framing aggregations and have similar statistically significant results in anticipation, trust, disgust, sadness, and fear words (Table A.7). Our analysis of linguistic shifts within the different helper therapy framings suggests that the “like you” target peer framing broadly elicits more positive emotion language and less negative emotion language over the more generic target peer framing in terms of total text produced within the framing intervention, pointing to potential benefits of the self-focused peer support framings beyond encouraging engagement with the helper therapy exercise.

## 6 Discussion

In this work, we estimate causal effects of different helper therapy exercise framings on supporting user engagement through randomized variation of 1) the intended “target peer” and 2) the motivating “rationale” for the exercise. Across user demographics, framings that center the exercise on the user (“self-focused” motivation and “someone like you” peer target) yield higher engagement compared to other framings. We now discuss conceptual implications, practical implications for DMH program design, and limitations alongside opportunities for future work.

### 6.1 Conceptual Implications

Our findings have implications for how peer support and helper therapy are understood in HCI. Peer-support systems are often framed as opportunities to help others [96], and altruistic motivations are frequently endorsed in volunteering contexts broadly [21, 38, 90–92]. Yet in our study, framings that emphasized one’s own needs were more motivating for helping behavior than framings that emphasized benefits to others. This pattern may reflect differences between self-reported motives and behavioral responses to framing, as well as characteristics of Mental Health America users, who are often early in their help-seeking journey [52]. Prior work on peer support also suggests that individuals early in recovery may not yet feel ready or able to help others [88], perhaps making self-focused framings a more accessible entry point for helper therapy. Findings may also be consistent with the increased self-focus among individuals experiencing mood disorders [41]. Self-focused framings may also reduce the cognitive burden associated with imagining the needs of an undefined audience. HCI work on “context collapse” shows that writing for indeterminate, heterogeneous online audiences can overwhelm contributors, leading to self-censorship [97]. Similar dynamics appear in peer-support tools such as Flip*Doubt, where users curated the negative thoughts they shared based on anticipated peer comprehension and responses [87]. Together, these perspectives suggest mechanisms through which self-focused framing may make helping feel more personally relevant and accessible, which future work can assess.

Beyond these conceptual mechanisms, our findings suggest that a framing difference, evaluated in a context of equipoise within an ongoing intervention, can change participation rates for helper therapy (Section 5.2). In this context, greater participation may have carry-on effects, as users’ messages can be used to help others or oneself (e.g., by sending the message to oneself later). To better understand these framings, it will be important to investigate these carry-on effects, including how receivers perceive messages produced under each framing. For example, we found greater positive emotion language in the context of self-focused framings (Section 5.4), and future work can investigate how support messages with emotional language are received. Prior work suggests that users are interested in receiving self-authored messages to motivate themselves [51], so comparing effects of self- and peer-authored message receiving could be a promising future direction.

### 6.2 Practical Implications for DMH Program Design

Our findings have practical implications for designing peer support systems. They suggest value in framing helping around benefits to the self and in prompting users to imagine themselves as the audience for their writing. The implications extend beyond explicit helper therapy or giving support, as many activities in peer-to-peer systems could be framed to benefit others, the self, or both. For example, sharing personal narratives or disclosing challenges can function as peer support by normalizing experiences and offering practical examples [13], and may also benefit the self through reflection and processing, similar to expressive writing [58]. Therefore, peer support systems offer many opportunities to frame participation as a pathway to engagement.

The increased efficacy of self-focused framing for encouraging user participation could be operationalized across different facets of DMH program design and deployment. Because our study focuses on a relatively high-effort, open-ended DMH exercise of writing support messages (as opposed to e.g., reading informational resources or completing structured questionnaires), our findings suggest that self-focused framing could be deployed to elicit more *active* user participation in exercises, which is a proposed mechanism of change in symptoms [34]. As individuals’ energy and motivation to engage with mental health self-management can fluctuate with symptoms [51], the framings could also be introduced at points of likely drop-off when user context suggests that they could benefit from encouragement. At the same time, DMH program designers need to be mindful of positioning users as “helpers” in ways that may not fit their current context. While many people seek out DMH tools because they want to build self-efficacy, energy and readiness to engage may still vary, and different exercise framings could resonate at one moment, yet could feel burdensome at another. Thus, it may be prudent to present self-focused framings selectively for higher-effort exercises rather than universally across all DMH exercises, or if the program has contextual information suggesting that a user may benefit from the framing, such as if they responded positively to the framing in the past through adaptive feedback, or if they identify with the demographic groups that are particularly receptive to these framings. Even when self-oriented framings perform best, there may also be benefits to using other-focused framings selectively, as variety and novelty can be important drivers of engagement, and individuals may potentially shift in their framing preferences as they spend more time in the program [49].

Our findings also have implications for online peer-support communities that are not exclusively DMH interventions. Platforms such as Koko (www.kokocares.org), TalkLife (www.talklife.com), and 7 Cups (www.7cups.com) provide peer-to-peer support opportunities for individuals with mental health conditions including depression and anxiety, and often provide guidelines and trainings to help peer supporters write more effective responses [66, 72, 89, 101]. Building on our findings, these materials could more explicitly coach helpers to emphasize shared connection for those seeking help, especially for new support seekers or those with minoritized identities, where “like-me” framings and shared commonalities can support trust and belonging. The form these materials take can be lightweight: for example, Koko has shown the value of providing brief signposting on how to effectively provide peer support [67], while other communities like TalkLife have integrated machine learning and natural language processing to provide feedback to supporters about effective peer-support messages [82]. This emphasis on shared connection also aligns with accounts of online community building that depend on users switching between roles of helping and being helped [74]. Brief self-focused helper framings could thus be be a way to encourage participation while still preserving possible role shifts in the community over time.

Beyond framing within a single DMH exercise, messaging interventions offer many opportunities to sustain engagement. Our findings suggest adjusting messaging to speak directly to users’ motivation. It may be possible to further personalize message framings to align with user preferences, such as through crowdsourcing [99]. Prior work has found that crowdworkers for mental health messages primarily produce “emotional support” content focusing on affirmation, reassurance, and inspiration [50], and presenting example messages that align with self-focused framing could prompt users to diversify and iterate on messages that resonate most with them. Our findings also show certain subgroups may benefit more from self-focused message framings (Section 5.3), suggesting value of assessing demographic characteristics or initial motivations on which to tailor messaging. To further improve tailoring, future work can investigate how motivations shift over time and use qualitative approaches to explore user motivations and engagement mechanisms. For example, individuals may become more involved in giving than receiving support as they spend time in a support community [35]. There is also likely to be individual-level variation in what messaging resonates, and personalization could be operationalized through adaptive experiments that learn which variants best support engagement for a given user [54] or allowing users to customize content (e.g., choosing preferred framings) [15]. Since peers produce diverse support messages [50], future work could apply data-driven approaches to help match supportive content to receivers most likely to benefit [65].

### 6.3 Limitations

A primary limitation of this work is that the relatively large (*n* = 3, 817) study sample voluntarily signed up for the program and may not be representative of the broader population of individuals experiencing mental health challenges. In particular, our sample has a high proportion of users who identify as women (75%) and LGBTQ+ (28%) relative to the U.S population. Users also had elevated psychological distress as measured by K10. Given the sample composition, our findings seem most applicable to a population experiencing mental health distress and willing to seek self-help.

Aspects of the deployed DMH program design also constrain the scope of our findings. The Small Steps intervention put all users in the role of helper, conceptualizing the exercise as helper therapy. Thus our analyses focus on the helper rather than the help-seeker who may later receive their message. Understanding which framings best support the helper may indirectly improve the support delivered by increasing helper engagement, but further work should also examine how messages produced under each framing are perceived by those who ultimately receive them so that both parties benefit. Additionally, the target peer framing focuses specifically on depression. However, users who are less familiar with depression may be unsure how to best support someone experiencing it. Future work could personalize prompts to users based on the specific mental health concerns they report.

We also acknowledge limitations in aspects of our study measurements. Our analysis focuses on short-term engagement, examining whether or not users responded to a single helper therapy writing task embedded in a longer-term 8-week DMH program. As sustained use drives the mental wellness benefits for users of these programs [28], follow-up work could examine longitudinal effects of helper therapy framings, particularly if the same users returned to the same exercise later in the program. As our analysis here focused on main effects of the interventions within demographic subgroups, future work could also investigate the interaction between the rationale and peer target framings for finer-grained effects for further personalization. Our linguistic analysis is restricted to users who responded and is strictly quantitative, making qualitative analysis of support message content a natural future direction. Ideally, we would also directly quantify mental health symptom change, but no measures beyond initial screening were collected by the program. Instead, we focus on engagement outcomes as a mechanism that facilitates the therapeutic effects intended by DMH tools [34].

## 7 Conclusion

Here we have studied how variations in the framing of a DMH helper therapy writing task may differentially support user engagement. We find causal evidence that self-focused framings are particularly effective in encouraging higher engagement rates within the overall study sample, with even larger effects of self-focused framings within BIPOC and LGBTQ+ identities. Our work presents real-world evidence of the effectiveness of different helper therapy framings, which can help inform the design of future DMH interventions to align with a diversity of identities.

## Figures and Tables

**Table 1:** Demographic characteristics of users participating in the Small Steps helper therapy writing exercise.

Variable	N	%	Variable	N or Mean ± SD	% or (n)
Gender Identity			Race		
Woman	2876	75.3%	White	2414	63.2%
Man	473	12.4%	Black	429	11.2%
Non-binary	107	2.8%	Multi-racial	359	9.4%
Self-describe	36	0.9%	Asian	165	4.3%
Missing	325	8.5%	American Indian	54	1.4%
			Native Hawaiian	12	0.3%
Transgender			Missing	384	10.1%
No	3398	89.0%			
Yes	81	2.1%	Hispanic		
Missing	338	8.9%	No	2881	75.5%
			Yes	549	14.4%
Sexual Orientation			Don’t know	46	1.2%
Heterosexual	2120	55.5%	Missing	341	8.9%
Bisexual	464	12.2%			
Asexual	178	4.7%			
Prefer not to answer	165	4.3%	Age (years)	35.94 ± 12.69	(n=3817)
Unsure	146	3.8%			
Homosexual	142	3.7%	K10 Score	33.72 ± 8.14	(n=3443)
Pansexual	140	3.7%	Missing	374	(9.8%)
Queer	72	1.9%			
Other	40	1.0%			
Missing	350	9.2%			

**Table 2:** Overall support message engagement rates within populations of analysis. The number of users sending support messages is shown in parentheses.

	Sent Support Message?
Entire Study Sample (n=3817)	19.5% (743)
LGBTQ+ Users (n=1071)	20.3% (217)
BIPOC Users (n=1019)	21.6% (220)

**Table 3:** Descriptives on message length in characters and token counts for users who sent a support message.

	Message Length		Token Counts	
Subgroup	Median [Q1, Q3]	Mean ± SD	Median [Q1, Q3]	Mean ± SD
Entire Study Sample	106.0 [51.0, 205.8]	170.8 ± 215.7	22.0 [10.0, 41.0]	34.2 ± 42.1
LGBTQ+ Users	120.0 [63.2, 239.2]	188.5 ± 227.8	24.0 [12.0, 45.8]	37.5 ± 43.4
BIPOC Users	108.0 [54.0, 214.0]	183.5 ± 238.4	23.0 [11.0, 42.0]	36.9 ± 46.3

**Table 4:** Causal effects of *rationale* framing on engagement with the helper therapy writing exercise.

	Others-focused Motivation vs No Rationale Sent	Self-focused Motivation vs No Rationale Sent	Self-focused Motivation vs Others-focused Motivation
Entire Study Sample	−2.0% [−5.0, 1.0]	+2.6% [−0.5, 5.8]	+4.6%* [1.6, 7.7]
LGBTQ+ Users	−0.4% [−6.0, 5.2]	+8.1%* [2.1, 14.1]	+8.6%* [2.5, 14.5]
BIPOC Users	−1.4% [−7.5, 4.6]	+2.6% [−3.8, 8.9]	+4.0% [−2.2, 10.2]

Cells indicate the change in engagement rate of the first framing over the second framing for each intervention comparison shown in the columns. Brackets indicate 95% confidence intervals. Bold values with asterisks * indicate statistically significant results at an *α* = 0.05 level after multiple comparisons correction.

**Table 5:** Causal effects of *target peer* framing on engagement with the helper therapy writing exercise.

	Support a Friend vs Support Someone Depressed	Support Someone Like You vs Support Someone Depressed	Support Someone Like You vs Support a Friend
Entire Study Sample	−0.3% [−3.4, 2.8]	−0.0% [−3.1, 3.0]	+0.2% [−2.9, 3.4]
LGBTQ+ Users	−1.3% [−7.1, 4.7]	−0.5% [−6.3, 5.3]	+0.7% [−5.3, 6.7]
BIPOC Users	+6.5% [0.6, 12.5]	+9.5%* [3.4, 15.5]	+2.9% [−3.7, 9.5]

Cells indicate the change in engagement rate of the first framing over the second framing for each intervention comparison shown in the columns. Brackets indicate 95% confidence intervals. Bold values with asterisks * indicate statistically significant results at an *α* = 0.05 level after multiple comparisons correction.

**Table 6:** Pairwise differences in NRC EmoLex emotion word category frequencies for *target peer* framings (change in percentage of total words).

Emotion Category	Support a Friend vs Support Someone Depressed	Support Someone Like You vs Support Someone Depressed	Support Someone Like You vs Support a Friend
Positive	+1.8% [−0.1, 3.5]	+3.3%* [1.5, 5.1]	+1.6% [−0.3, 3.6]
Anticipation	+1.6% [0.2, 3.0]	+3.1%* [1.8, 4.5]	+1.5% [0.1, 2.9]
Trust	+0.6% [−0.5, 1.7]	+1.9%* [0.7, 3.0]	+1.3% [0.0, 2.6]
Joy	+0.8% [−0.5, 2.1]	+1.4% [−0.0, 2.8]	+0.6% [−0.8, 1.9]
Surprise	−0.2% [−1.2, 0.8]	−0.3% [−1.2, 0.6]	−0.1% [−1.2, 0.9]
Negative	−0.5% [−1.9, 1.1]	−1.4% [−2.7, 0.1]	−0.9% [−2.3, 0.7]
Anger	−0.7% [−1.7, 0.5]	−1.6%* [−2.6, −0.6]	−1.0% [−2.0, 0.0]
Disgust	−0.7% [−1.6, 0.2]	−1.9%* [−2.8, −0.9]	−1.2% [−2.0, −0.3]
Sadness	−1.3% [−2.5, 0.0]	−2.2%* [−3.3, −1.0]	−0.9% [−2.0, 0.3]
Fear	−1.4% [−2.5, −0.3]	−2.4%* [−3.5, −1.1]	−0.9% [−2.0, 0.1]

Brackets indicate 95% confidence intervals. Bold values with asterisks * indicate statistically significant results at an *α* = 0.05 level after multiple comparisons correction.

## CCS Concepts

• Human-centered computing → Empirical studies in HCI; Field studies.

## A Appendix

### A.1 Variable Definitions

We consider an individual to be LGBTQ+-identified if they made any of the following responses in the voluntary demographics survey:

- Gender identity: non-binary or self-describe
- Transgender: yes
- Sexual orientation: bisexual, asexual, homosexual, pansexual, queer, or other

We consider an individual to be BIPOC-identified if they made any of the following responses in the voluntary demographics survey:

- Race: Black, Multi-racial, Asian, American Indian, Native Hawaiian

### A.2 Language Result Details

As word dictionaries require a minimum level of text to produce stable measurements [16, 63], instead of analyzing per-responder text we aggregate messages within the different helper therapy framing variations. This alters the interpretation from a per-user quantity to a “total text emitted” per helper therapy framing, which upweights user responses that are longer. color This per-framing aggregation also maintains the causal interpretation of the framing intervention, as aggregating per message necessarily conditions the results only on those who engage with the exercise. Since the text is aggregated within framings we use permutation testing [33] and 1,000 bootstrapped draws from user support messages within a framing to generate p-values and confidence intervals for the measured differences of emotion language between framings (Table 6, Table A.8).

As a robustness check, we also perform all of our linguistic analysis aggregating across per-responder text. We analyze messages that meet a 20 token cutoff with stop-words removed to balance the stability of the per-user linguistic measurements [63] with the overall sample size of messages that meet the token limit. The per-message LIWC language descriptives closely agree with the total word descriptives (Table A.5 and Table A.6). t-tests are performed to generate p-values and confidence intervals for the measured differences of emotion language between framings on a per-message level (Table A.7, Table A.9).

**Table A.1:** Full system messages for the target peer and rationale framing variations.

Intervention	System Text Message
Self-Focused Rationale	“The “Helper therapy” principle says that helping others can help you as well”
Other-Focused Rationale	“If you have experienced depression before, you might be able to help others in a way that professionals can’t”
“Someone Depressed” Peer Target	“Please help us write a short message that might motivate someone who feels depressed. What would you say to help them to get through a bad day?”
“Someone Like You” Peer Target	“Please help us write a short message that might motivate someone like you if they felt depressed. What would you say to help them to get through a bad day?”
“A Friend” Peer Target	“Please help us write a short message that might motivate a friend who feels depressed. What would you say to help them to get through a bad day?”

**Table A.2:** Example user support messages sent as part of the helper therapy writing exercise across different individual conversations.

Support Message
“Just wanted to stop by and say, I’m with you! You have my permission to feel 100% how you feel because they are your feelings. No one has a right to tell you what to feel or how long to feel it. I stand with you in support of your feelings and in support of you taking as long as you need to get through this” “Though you feel like you haven’t accomplished anything today remember waking up is actually a victory and you are enough and loved by so many even though you may not realize. I’m here for you if you need a friend” “Progress over perfection is important to keep in mind. Hard days end and if all you did was survive then that’s enough. Getting through the hard days gives you a chance for better days” “You’ve touched so many people’s lives.” “It may seem dark right now, but I promise better days are coming. Just gotta hold on and hang on.” “Find one thing to be grateful for… often, when we go down a dark path, we do not see the rainbow on the other side. So find one thing than another and another until you see the silver lining in the darkness.” “You matter the world needs you” “You are not alone. Reach out for help, even when you don’t want to. Or, just be kind to yourself and know it’s ok to have a bad period. This too shall pass”

**Table A.3:** Covariate balance among the *peer target* randomized prompts.

	Support Someone Depressed	Support a Friend	Support Someone Like You
Age (years): mean ± SD	36.07 ± 12.70	36.29 ± 12.84	35.48 ± 12.54
K10 Score: mean ± SD	33.57 ± 8.01	33.83 ± 8.28	33.77 ± 8.14
Gender Identity: % (count)			
Woman	82.6% (1035)	81.7% (881)	82.7% (960)
Man	13.6% (170)	13.8% (149)	13.3% (154)
Non-binary	3.2% (40)	3.5% (38)	2.5% (29)
Self-describe	0.6% (8)	0.9% (10)	1.6% (18)
LGBTQ+ Identity: % (count)			
No	71.4% (970)	73.0% (871)	71.5% (905)
Yes	28.6% (389)	27.0% (322)	28.5% (360)
BIPOC Identity: % (count)			
No	73.1% (994)	73.3% (875)	73.4% (929)
Yes	26.9% (365)	26.7% (318)	26.6% (336)

**Table A.4:** Covariate balance among the *rationale* randomized prompts.

	No Rationale Sent	Others-focused Motivation	Self-focused Motivation
Age (years): mean ± SD	35.85 ± 12.60	36.25 ± 12.73	35.73 ± 12.74
K10 Score: mean ± SD	33.66 ± 8.09	33.70 ± 8.12	33.80 ± 8.20
Gender Identity: % (count)	82.0% (918)	82.1% (978)	82.9% (980)
Woman			
Man	13.9% (155)	13.7% (163)	13.1% (155)
Non-binary	3.1% (35)	3.1% (37)	3.0% (35)
Self-describe	1.0% (11)	1.1% (13)	1.0% (12)
LGBTQ+ Identity: % (count)	71.1% (875)	72.2% (934)	72.5% (937)
No			
Yes	28.9% (356)	27.8% (359)	27.5% (356)
BIPOC Identity: % (count)	72.5% (892)	73.1% (945)	74.3% (961)
No			
Yes	27.5% (339)	26.9% (348)	25.7% (332)

**Table A.5:** Word frequencies as a percentage of the total support message text sent for select LIWC hierarchical categories.

Category	% of total words
First Person Singular (I)	3.71%
Second Person (You)	7.35%
First Person Plural (We)	0.55%
All or None Thinking	1.85%
Insight	4.29%
Causation	1.74%
Positive Emotional Tone	4.97%
Negative Emotional Tone	2.33%
Positive Emotion	1.72%
Negative Emotion	1.55%
Anxiety	0.19%
Anger	0.02%
Sadness	0.75%
Prosocial Behavior	1.11%
Politeness	0.18%
Conflict	0.14%
Moral Concerns	0.21%
Communication	1.23%
Wellness	0.18%
Mental Health	0.42%
Past Focus	2.06%
Present Focus	9.31%
Future Focus	2.9%

**Table A.6:** Word frequencies per message as a percentage of the total words for select LIWC hierarchical categories among messages that meet a 20 token threshold.

Category	Mean % ± SD of total words
First Person Singular (I)	3.17% ± 4.73
Second Person (You)	7.66% ± 5.52
First Person Plural (We)	0.48% ± 1.63
All or None Thinking	1.89% ± 2.59
Insight	4.32% ± 3.65
Causation	1.8% ± 2.34
Positive Emotional Tone	4.92% ± 3.82
Negative Emotional Tone	2.38% ± 2.95
Positive Emotion	1.78% ± 2.26
Negative Emotion	1.65% ± 2.42
Anxiety	0.16% ± 0.65
Anger	0.02% ± 0.28
Sadness	0.72% ± 1.58
Prosocial Behavior	1.01% ± 1.73
Politeness	0.17% ± 0.67
Conflict	0.12% ± 0.57
Moral Concerns	0.19% ± 0.68
Communication	1.19% ± 1.92
Wellness	0.15% ± 0.73
Mental Health	0.37% ± 1.1
Past Focus	1.74% ± 2.85
Present Focus	9.57% ± 4.89
Future Focus	3.01% ± 3.51

**Table A.7:** Pairwise differences in NRC EmoLex emotion word category frequencies for per-message *peer target* framings (change in percentage of words) among messages that meet a 20 token threshold.

Emotion Category	Support a Friend vs Support Someone Depressed	Support Someone Like You vs Support Someone Depressed	Support Someone Like You vs Support a Friend
Positive	+5.7% [1.0, 10.0]	+4.5% [0.0, 9.0]	−1.3% [−6.0, 3.0]
Anticipation	+0.0% [−3.0, 3.0]	**+3.6%* [1.0, 7.0]**	+3.5% [1.0, 6.0]
Trust	+2.2% [−1.0, 5.0]	**+3.5%* [1.0, 6.0]**	+1.4% [−1.0, 4.0]
Joy	−0.3% [−3.0, 3.0]	+1.3% [−2.0, 5.0]	+1.7% [−1.0, 5.0]
Surprise	−0.1% [−2.0, 2.0]	+1.2% [−1.0, 3.0]	+1.3% [−1.0, 3.0]
Negative	−1.1% [−5.0, 2.0]	−3.5% [−7.0, −0.0]	−2.4% [−5.0, 1.0]
Anger	−1.0% [−3.0, 1.0]	−1.6% [−4.0, 1.0]	−0.6% [−3.0, 1.0]
Disgust	−1.1% [−3.0, 1.0]	**−2.2%* [−4.0, −0.1]**	−1.1% [−3.0, 0.0]
Sadness	−2.5% [−5.0, 0.0]	**−4.3%* [−7.0, −2.0]**	−1.8% [−4.0, 1.0]
Fear	−2.9% [−6.0, 0.0]	**−4.5%* [−7.0, −2.0]**	−1.6% [−4.0, 1.0]

Brackets indicate 95% confidence intervals, and bold values with asterisks * indicate statistically significant results at an *α* = 0.05 level after multiple comparisons correction.

**Table A.8:** Pairwise differences in NRC EmoLex emotion word category frequencies for *rationale* framings (change in percentage of total words). Brackets indicate 95% confidence intervals.

Emotion Category	Others-focused Motivation vs No Rationale Sent	Self-focused Motivation vs No Rationale Sent	Self-focused Motivation vs Others-focused Motivation
Positive	−1.6% [−3.5, 0.5]	−0.8% [−2.7, 1.1]	+0.7% [−1.2, 2.7]
Anticipation	−2.0% [−3.6, −0.5]	−1.5% [−2.8, −0.2]	+0.5% [−1.1, 2.1]
Trust	−0.0% [−1.4, 1.4]	+0.2% [−1.0, 1.5]	+0.2% [−1.1, 1.6]
Joy	−0.6% [−1.9, 0.7]	+0.1% [−1.2, 1.4]	+0.7% [−0.8, 2.2]
Surprise	−0.7% [−1.7, 0.2]	−0.2% [−1.2, 0.7]	+0.5% [−0.4, 1.5]
Negative	+1.9% [0.4, 3.4]	+1.0% [−0.4, 2.3]	−0.9% [−2.5, 0.7]
Anger	+0.9% [−0.3, 2.1]	+0.1% [−0.9, 1.1]	−0.8% [−1.9, 0.4]
Disgust	+0.4% [−0.5, 1.3]	+0.1% [−0.8, 1.0]	−0.3% [−1.2, 0.7]
Sadness	+1.8% [0.6, 3.1]	+1.2% [−0.1, 2.4]	−0.7% [−1.9, 0.6]
Fear	−0.1% [−1.4, 1.1]	−0.1% [−1.2, 1.0]	−0.0% [−1.1, 1.2]

**Table A.9:** Pairwise differences in NRC EmoLex emotion word category frequencies for per-message *rationale* framings (change in percentage of words) among messages that meet a 20 token threshold. Brackets indicate 95% confidence intervals.

Emotion Category	Others-focused Motivation vs No Rationale Sent	Self-focused Motivation vs No Rationale Sent	Self-focused Motivation vs Others-focused Motivation
Positive	−1.2% [−6.0, 4.0]	−0.7% [−5.0, 4.0]	+0.5% [−4.0, 5.0]
Anticipation	−2.6% [−6.0, 0.0]	−0.3% [−3.0, 2.0]	+2.3% [−0.0, 5.0]
Trust	+0.3% [−3.0, 3.0]	+1.0% [−1.0, 3.0]	+0.7% [−2.0, 3.0]
Joy	−0.8% [−4.0, 2.0]	+1.4% [−2.0, 5.0]	+2.2% [−1.0, 5.0]
Surprise	−1.6% [−4.0, 0.0]	+0.0% [−2.0, 2.0]	+1.7% [−0.0, 3.0]
Negative	+2.9% [−0.0, 6.0]	−1.4% [−4.0, 2.0]	−4.3% [−8.0, −1.0]
Anger	+1.8% [−1.0, 4.0]	−0.4% [−2.0, 2.0]	−2.1% [−4.0, −0.0]
Disgust	+1.0% [−1.0, 3.0]	−0.6% [−2.0, 1.0]	−1.6% [−3.0, 0.0]
Sadness	+2.7% [−0.0, 6.0]	+1.4% [−1.0, 4.0]	−1.2% [−4.0, 2.0]
Fear	−1.1% [−4.0, 2.0]	−1.3% [−4.0, 2.0]	−0.2% [−3.0, 2.0]
